# Supplementary material for: The Systems Biology Research Tool: evolvable open-source software
Source: BMC Syst Biol. 2008 Jun 29;2:55. doi: 10.1186/1752-0509-2-55 (PMC2446383; doi:10.1186/1752-0509-2-55)
Supplement: Additional file 1 — SBRT Archive. An archive of the current version of the Systems Biology Research Tool. [file 1752-0509-2-55-S1.zip › sbrt-1.4.0/doc/developers_guide/api/sbrt/kernel/fba/comp/FluxCapException.html]

FluxCapException


|  |  |  |
| --- | --- | --- |
| **Overview** \| **Package** \| **Class** \| **Use** \| **Tree** \| **Deprecated** \| **Index** \| **Help** | |  |
| **PREV CLASS**   **NEXT CLASS** | **FRAMES**    **NO FRAMES**     **All Classes** |  |
| SUMMARY: NESTED \| FIELD \| CONSTR \| METHOD | DETAIL: FIELD \| CONSTR \| METHOD |  |


---


## sbrt.kernel.fba.comp Class FluxCapException

```
java.lang.Object
  java.lang.Throwable
      java.lang.Exception
          java.lang.RuntimeException
              sbrt.kernel.fba.comp.FluxCapException
```

**All Implemented Interfaces:**: java.io.Serializable

---

``` public class FluxCapException extends java.lang.RuntimeException ```

This class is used to indicate that a flux cap is invalid. A flux cap is
invalid if it does not cap the flux of the intended reaction.

**Author:**
:   This class was written and documented by Jeremiah Wright
    while in the Wagner lab.

**See Also:**: Serialized Form

---

**Constructor Summary**

`FluxCapException(MathExpr fluxCap,
java.lang.String reactionName)`
  
          Constructs a new flux cap exception.

`FluxCapException(MathExpr fluxCap,
java.lang.String reactionName,
java.lang.Throwable cause)`
  
          Constructs a new flux cap exception.


| **Method Summary** | |
| --- | --- |
| `MathExpr` | `getFluxCap()`             Returns the invalid flux cap. |
| `java.lang.String` | `getReactioName()`             Returns the name of the reaction whose flux was intended to be capped. |

**Methods inherited from class java.lang.Throwable**

`fillInStackTrace, getCause, getLocalizedMessage, getMessage, getStackTrace, initCause, printStackTrace, printStackTrace, printStackTrace, setStackTrace, toString`

**Methods inherited from class java.lang.Object**

`clone, equals, finalize, getClass, hashCode, notify, notifyAll, wait, wait, wait`

**Constructor Detail**

### FluxCapException

```
public FluxCapException(MathExpr fluxCap,
                        java.lang.String reactionName,
                        java.lang.Throwable cause)
```

:   Constructs a new flux cap exception.

    **Parameters:**: `fluxCap` - the invalid flux cap.: `reactionName` - the name of the reaction whose flux was intended to be capped.: `cause` - the cause.

---


### FluxCapException

```
public FluxCapException(MathExpr fluxCap,
                        java.lang.String reactionName)
```

:   Constructs a new flux cap exception.

    **Parameters:**: `fluxCap` - the invalid flux cap.: `reactionName` - the name of the reaction whose flux was intended to be capped.


**Method Detail**

### getFluxCap

```
public MathExpr getFluxCap()
```

:   Returns the invalid flux cap.

    :   **Returns:**: the invalid flux cap.

---


### getReactioName

```
public java.lang.String getReactioName()
```

:   Returns the name of the reaction whose flux was intended to be capped.

    :   **Returns:**: the name of the reaction whose flux was intended to be capped.


---


|  |  |  |
| --- | --- | --- |
| **Overview** \| **Package** \| **Class** \| **Use** \| **Tree** \| **Deprecated** \| **Index** \| **Help** | |  |
| **PREV CLASS**   **NEXT CLASS** | **FRAMES**    **NO FRAMES**     **All Classes** |  |
| SUMMARY: NESTED \| FIELD \| CONSTR \| METHOD | DETAIL: FIELD \| CONSTR \| METHOD |  |


---
